# A meta-analysis and systematic review on subtypes of gastric intestinal metaplasia and neoplasia risk

**DOI:** 10.1186/s12935-021-01869-0

**Published:** 2021-03-17

**Authors:** Ning Wei, Mengyue Zhou, Siyu Lei, Zhiheng Zhong, Ruihua Shi

**Affiliations:** 1 Medical School of Southeast University, Nanjing, 210009 China; 2 Department of Gastroenterology, Southeast University Affiliated Zhongda Hospital, No. 87 Dingjiaqiao, Nanjing, 210009 China

**Keywords:** Subtypes, Intestinal metaplasia, Gastric cancer, Dysplasia, Meta-analysis

## Abstract

**Background:**

Gastric intestinal metaplasia (GIM) is a significant risk factor for gastric cancer. Risk of gastric cancer/dysplasia between complete intestinal metaplasia (CIM) and incomplete intestinal metaplasia (IIM) was controversial. Our study aimed to pool relative risk (RR) of cancer/dysplasia of IIM compared with CIM in GIM patients.

**Methods:**

PubMed, EMBASE, Cochrane Library and Web of Science were searched for studies concerning cancer/dysplasia in GIM patients. Random-effects or fixed-effects model was utilized for pooling RR. Sensitivity and publication bias analyses were conducted. Stability of results would be evaluated in case of publication bias.

**Results:**

12 studies were included. Compared with CIM, pooled RR of cancer/dysplasia in IIM patients was 4.48 (95% CI 2.50–8.03), and the RR was 4.96 (95% CI 2.72–9.04) for cancer, and 4.82 (95% CI 1.45–16.0) for dysplasia. The pooled RR for cancer/dysplasia in type III IM was 6.27 (95% CI 1.89–20.77) compared with type II + I IM, while it was 5.55 (95% CI 2.07–14.92) compared with type II IM. Pooled RR between type II IM and type I IM was 1.62 (95% CI 1.16–2.27). Subgroup analyses showed that IIM was associated with a higher risk of gastric cancer/dysplasia in Western population (pooled RR = 4.65 95% CI 2.30–9.42), but not in East Asian population (pooled RR = 4.01 95% CI 0.82–19.61).

**Conclusions:**

IIM was related to a higher risk of cancer/dysplasia compared with CIM. Risk of developing cancer/dysplasia from type I, II, and III intestinal metaplasia increased gradually.

**Supplementary Information:**

The online version contains supplementary material available at 10.1186/s12935-021-01869-0.

## Background

According to Correa’s gastric cancer model, gastric intestinal metaplasia (GIM) is a significant risk factor for gastric cancer [1]. GIM was found in 25.3% of patients with dyspepsia and 100% of patients with intestinal-type gastric cancer [2]. Around 1 in every 39 patients with GIM would progress into gastric cancer within 20 years [3], which was similar to the result of De Vries et al. [4] an annual incidence of gastric cancer of 0.25%. During patients undergoing routine endoscopy, the prevalence of GIM ranged from 13.8 to 19% in Europe [5, 6], 37% in Japan and 29% in China [7], which necessitates the further identification of high-risk patients among GIM patients. Some markers, such as score of operative link for gastric intestinal metaplasia assessment (OLGIM) [8], endoscopic grading of gastric intestinal metaplasia (EGGIM) [9, 10] and family history of gastric cancer [11], have been recommended for identifying those high-risk patients. There are three subtypes of GIM, with type I termed “complete IM” (CIM) and types II and III named “incomplete IM” (IIM). In some studies [12–14], patients with IIM were at higher risk for gastric cancer than those with CIM, which was inconsistent with other studies [15–20]. Therefore, additional studies are required before IIM can be used to distinguish patients at higher risk for gastric cancer [10]. In a subgroup analysis of the meta-analysis conducted by Shao et al. [1], IIM (pooled OR = 59.48, 95% CI 4.33–20.78) was associated with a higher risk of gastric cancer than CIM (pooled OR = 51.55, 95% CI 0.91–2.65) in patients with GIM. But the risk of malignant transformation of IIM compared with CIM was not clarified. In view of these controversies, our study aimed to pool the relative risk (RR) of cancer/dysplasia of IIM compared with CIM in patients with GIM. We also compared the predictive ability among 3 subtypes of intestinal metaplasia.

## Methods

### Search strategy

A literature search in online medical databases including PubMed, EMBASE, Cochrane Library, and Web of Science was performed by two authors (M.Z. and W.N.) independently to identify relevant studies (published until March 2020) on the incidence rate of cancer/dysplasia in patients with specific subtypes of GIM. Besides, we also checked the reference lists of relevant review articles and included studies to find any other eligible articles. Search strategy was based on the following terms and keywords: (“IM” OR “intestinal metaplasia”) AND (((“gastric” OR “stomach”) AND ((“cancer” OR “adenocarcinoma” OR “tumor” OR “carcinoma” OR “neoplasm”)) OR “dysplasia”) AND (“subtype” OR “variant” OR “type I”)).

### Inclusion and exclusion criteria

Two authors (M.Z. and N.W.) independently screened articles that met the following inclusion criteria: (1) compared with CIM, odds ratio, relative risk or hazard ratio with their 95% confidence interval (95% CI) (or data to calculate them) of cancer/dysplasia were reported in patients with IIM (2) there were cases of cancer/dysplasia in the cohort study. In vitro or animal studies, seminar reports, case reports, case series, and duplicate publications were excluded.

### Data extraction

Two reviewers (M.Z. and N.W.) independently conducted the data extraction procedure, and a third investigator (Y.L.) would resolve the inconsistency. Information including first author, publication year, study design, country of origin, sample size, duration of follow-up in cohort studies, risk estimates and adjusted factors, was extracted from each study.

### Quality assessment

The quality of each included study was assessed by 2 reviewers (M.Z. and N.W.) according to the Newcastle–Ottawa Scale (NOS) [21]. Article with total NOS score < 7 were rated as low-quality, and > 6 as high-quality studies.

### Statistical analysis

Relative result was pooled using either a random or fixed effects model on the basis of the result of heterogeneity analysis. Q and I^2^ statistics was used to evaluate the heterogeneity of our study, and p < 0.05 or I^2^ > 50% indicated significant heterogeneity [22]. The primary aim of our meta-analysis was to investigate RR of cancer/dysplasia in IIM when compared with CIM. We also conducted subgroup and sensitivity analyses to explore source of heterogeneity and to evaluate the pooled RR in 3 different subtypes of GIM (type III vs. II; type II vs. I; type III vs II + I), study design (cohort or case–control), district (East Asia or the West). Publication bias risk was evaluated by Egger’s test and funnel chart. If there was publication bias, we would evaluate the stability of the results by trim and fill method [23, 24]. All analyses were performed by the Stata software (V.15.0; Stata Corp, College Station, TX), and p values < 0.05 was considered significant. For the low morbidity of cancer/dysplasia, odds ratio and hazard ratio yield similar estimates of rate ratio in practice [25, 26].

## Results

### Characteristics of the included studies

**Fig. 1:**
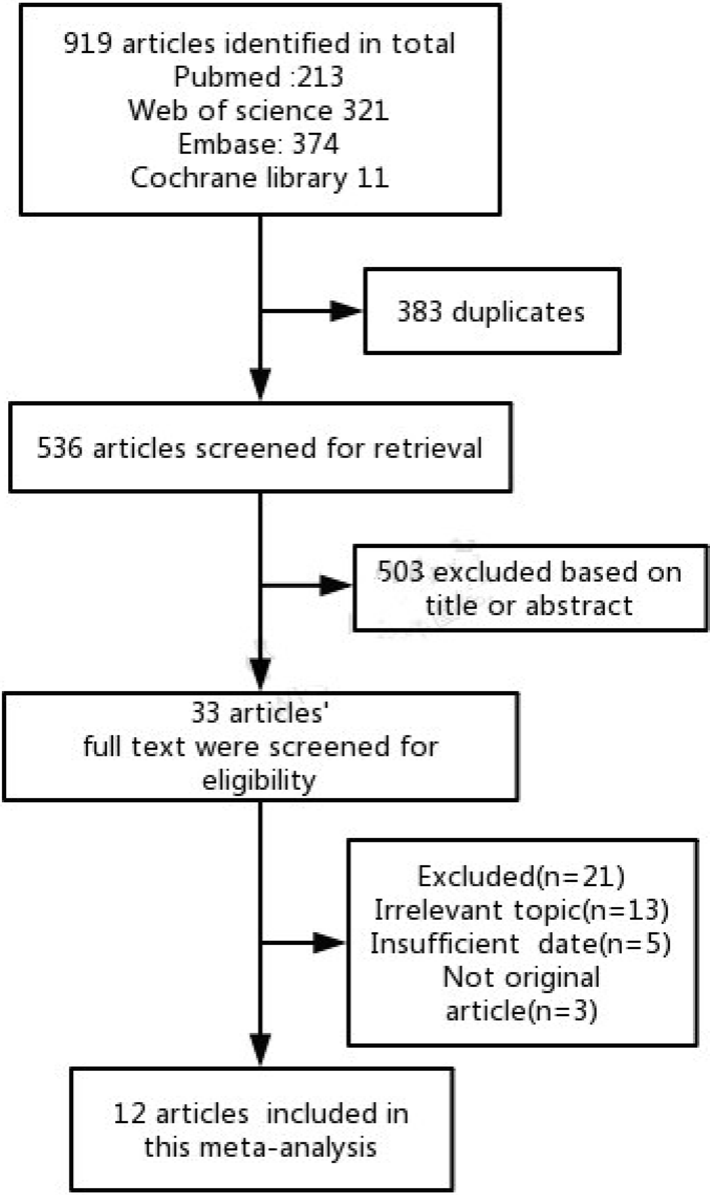
Flow diagram of the selection process

A total of 919 articles was collected initially from PubMed, Embase, Web of Science and Cochrane Library, of which 33 were potentially relevant reports for further review. And then 21 studies were excluded further for the following reasons: didn’t involve the risk of cancer/dysplasia of specific subtype of GIM (n = 13); lack of data (n = 5); not original articles (n = 3). As shown in Fig. 1, 12 studies were finally included in this meta-analysis.

Table 1 showed the characteristics of the included studies. 11 studies involved the cancer risk of IIM, 4 studies involved the dysplasia risk. Most of the included studies were conducted in the West (n = 8), 3 in East Asia, and one in Colombia [27]. During the included studies, 7 were cohort studies, while 5 were case–control studies. Each study included men and women. Most biopsy protocols included both the antrum and corpus. The main method to distinguish subtypes was using Alcian blue pH 2.5/periodic acid Schiff (AB-PAS) and high iron-diamine/Alcian blue pH 2.5 (HID/AB).

**Table 1:** Characteristics of the 12 included studies

Authors	Years	Country	Adjusted factors	Number of cancer/dysplasia/GIM	Design	Follow-up time (years)	Age (years) ± SD^a^	NOS Score	Biopsy sampling protocols	Methods for discrimination of subtypes
Cassaro [28]	2000	Italy	None	–/59/26	Case–c ontrol	–	48.6 ± 14.4 (cancer) 42.3 ± 11.5 (control)	7	5 from antrum; 6 from corpus; 1 from cardia	AB-PAS; HID/AB
González [29]	2010	Spain	None	–/17/175	Cohort	Mean 12.8	50.0 ± 12.2	7	2 from antrum; 1 from incisura angularis; 2 from corpus	AB-PAS; HID/AB
González [14]	2015	Spain	Sex, age, smoking, family history, use of NSAID	27/22/418	Cohort	Mean 12	52.17 ± 10.13	7	At least 3 fragments, including both antrum and corpus	AB-PAS; HID/AB
Mera [27]	2017	Colombia	OLGA and OLGIM systems	–	Cohort	Up to 16	51 ± 9	8	2 from antrum; 1 from incisura angularis; 1 from corpus	AB-PAS; HID/AB; H&E staining
Pittayanon [30]	2017	Thailand	Hp, family history, smoking, drinking, preservative food consumption	3/3/85	Cohort	Mean 4.05	63 ± 13.3	8	Targeted biopsies from suspicious lesions under NBI	H&E staining
Rothery [31]	1985	UK	None	17/52/420	Case–control	–	59 ± 16	8	–	AB-PAS; HID/AB
Silva [17]	1986	Portuguese	None	–/72/172	Case–control	–	28–86 (range)	7	2–10 biopsies from antrum, corpus, and incisura angularis according to endoscopic findings	AB-PAS; HID/AB
Ramesar [32]	1987	Scotland	None	–/2/42	Cohort	10 to 11	66 ± 9 (11–5) (type I) 64 ± 3 (12–9) (type II) 65 ± 4 (11–6) (type III)	6	–	AB-PAS; HID/AB
Silva [33]	1990	UK	None	6/1/116	Cohort	Up to 6 year	–	8	1 from antrum (chronic gastritis group) or ulcers (ulcer group); additional biopsy specimens were taken if necessary	AB-PAS; HID/AB
Filipe [34]	1994	SLOVENIA	None	–/26/964	Cohort	Up to 20 year	–	7	2 from antrum; 1 from corpus	AB-PAS; HID/AB
Wu [35]	1998	China	None	–/90/49	Case–control	–	–	8	2 from antrum; 1 from incisura angularis; 2 from corpus	HID/AB; H&E staining
Kang [20]	2009	Korea	None	–/391/195	Case–control	–	57.7 ± 13.5	7	2 from antrum; 2 from corpus	AB-PAS; HID/AB

^a^Age distribution of all subjects in related studies

*AB-PAS* Alcian blue pH 2.5/periodic acid Schiff; *HID/AB* high iron-diamine/Alcian blue pH 2.5; *H&E* haematoxylin and eosin; *NBI* narrow-band imaging

### Gastric cancer/dysplasia risk among the patients with incomplete intestinal metaplasia

**Fig. 2:**
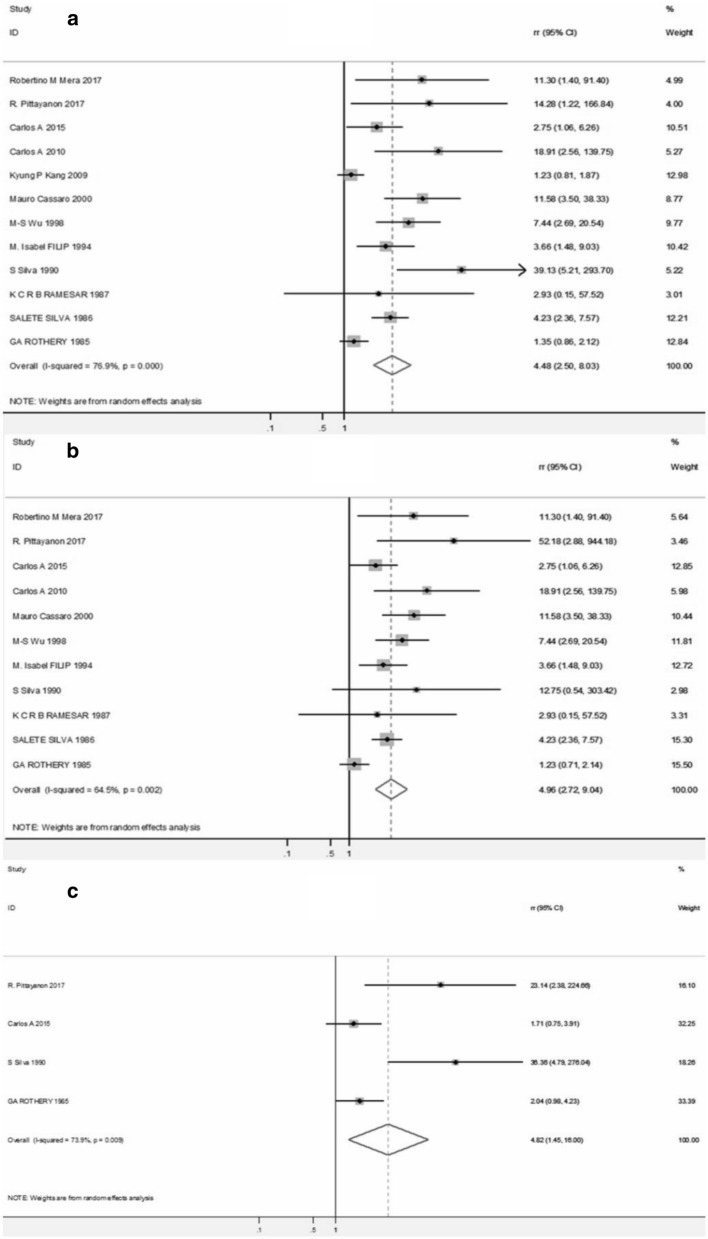
Forest plots for cancer/dysplasia (**a**), cancer (**b**), dysplasia (**c**) risk among patients with IIM when compared with CIM

Compared with CIM, the pooled RR of cancer/dysplasia risk among the patients with IIM was 4.48 (95% CI 2.50–8.03) with significant heterogeneity (I^2^ = 76.9%, p < 0.001) (Fig. 2a), indicating a higher cancer/dysplasia risk among the patients with IIM. Besides, for gastric cancer, compared with CIM, the pooled RR for patients with IIM was 4.96 (95% CI 2.72–9.04) (Fig. 2b). And incomplete IM was also associated with a significantly higher risk of dysplasia (pooled RR = 4.82, 95% CI 1.45–16.0) (Fig. 2c).

### Subgroup and sensitivity analyses

**Fig. 3:**
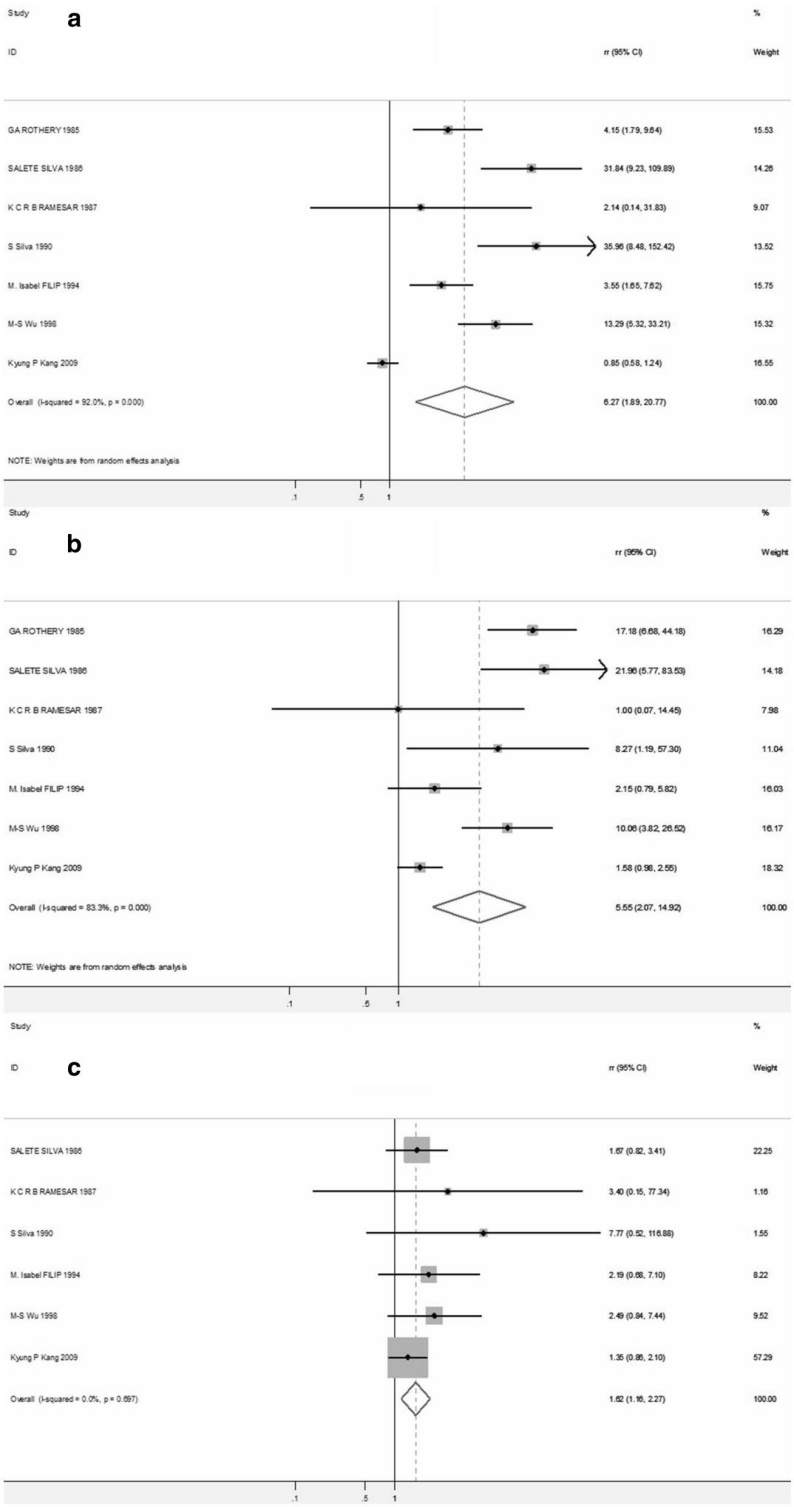
Forest plots for cancer/dysplasia risk among patients with type III IM when compared with type II + I IM (**a**), and with only type II IM (**b**). And cancer/dysplasia risk among patients with type II IM when compared with type I IM (**c**)

When it came to type III vs. II & I, type III vs. II, and type II vs. I, the RR of cancer/dysplasia were also performed respectively. The pooled RR for type III was 6.27 (95% CI 1.89–20.77) when compared with type II + I, while it was 5.55 (95% CI 2.07–14.92) when compared with only type II. The initial pooled RR between type II and I was 1.30 (95% CI 0.97–1.74) (Additional file 1: Figure S3), however 1 study [28] was excluded after sensitivity analysis (Additional file 1: Figure S4, Table S4), and the pooled RR of the remaining 6 studies was 1.62 (95% CI 1.16–2.27) (Fig. 3). Subgroup analyses were also performed according to study design and country of origin, as shown in Table 2. IIM was associated with a higher gastric cancer/dysplasia risk both in cohort studies (pooled RR = 5.05, 95% CI 2.07–14.92) and case–control studies (pooled RR = 3.15 95% CI 1.48–6.73). Moreover, pooled RR for the association between IIM and gastric cancer/dysplasia risk was significant in western countries (pooled RR = 4.65 95% CI 2.30–9.42), rather than East Asia (pooled RR = 4.01 95% CI 0.82–19.61).

**Table 2:** Subgroup analyses of GIM and risk of cancer/dysplasia

Group	No. of studies	Pooled RR (95% CI)	Heterogeneity	
			I^2^ (%)	P
Subtype				
III vs. II + I	7	6.27 (1.89–20.77)	92.0	< 0.001
III vs. II	7	5.55 (2.07–14.92)	83.3	< 0.001
II vs I^a^	6	1.62 (1.16–2.27)	0.0	0.697
Design				
Cohort	7	5.05 (2.96–8.63)	34.9	0.162
Case control	5	3.15 (1.48–6.73)	86.3	< 0.001
Country of origin				
East Asia	3	4.01 (0.82–19.61)	84.9	< 0.001
Western countries	8	4.65 (2.30–9.42)	74.8	< 0.001

^a^Results of the remaining 6 studies after excluding 1 study [31] by sensitivity analysis

Besides the result of type II vs. I IM, which was discussed above, the stability of the outcome was also confirmed by the sensitivity analysis (Additional file 1: Figure S1, Table S1).

### Publication bias

For the risk of cancer/dysplasia of IIM versus CIM, funnel plot and Egger’s test suggested that publication bias may exist (P value of Egger’s test = 0.002) (Additional file 1: Table S2, Figure S2). The results of trim and fill method showed that the conclusion was still significant after adding 5 potential missing studies [pooled RR = 2.664 (95% CI 1.546–4.591)] (Additional file 1: Table S3). Besides, the funnel plots showed that asymmetric studies were mostly those with small samples, and those with larger samples were basically symmetric, which further verified the reliability of the conclusion. Therefore, we can still conclude that IIM meant a higher risk of cancer/dysplasia than CIM.

## Discussion

At present, the predictive value of IIM for cancer/dysplasia is still controversial. Some studies still question the predictive value of GIM subtypes for neoplasia [15–18]. Therefore, the British Society of Gastroenterology (BSG) and Management of epithelial precancerous conditions and lesions in the stomach (MAPS II) [10, 36] both suggested that more relevant evidence was needed before further clinical application. Our study was the first meta-analysis on the cancer/dysplasia risk among patients with IIM compared with CIM. We found that among patients with GIM, patients with IIM had a significantly higher risk of developing cancer (pooled RR = 4.96 95% CI 2.72–9.04), dysplasia (pooled RR = 4.82 95% CI 1.45–16.0), and cancer/dysplasia (pooled RR = 4.48 95% CI 2.50–8.03) than those with CIM. Thus, the emergence of IIM had a certain predictive value of the occurrence of cancer/dysplasia. A study performed by Craanen et al. [37] found that type III IM was strongly associated with intestinal-type carcinoma but not with benign lesions (P < 0.01) or diffuse-type carcinoma. However, we did not pool the risk for intestinal-type cancer and diffuse-type gastric cancer respectively for lack of relative data, which needed further research.

In both study types included in the current study (cohort and case–control studies), patients had IIM were at higher risk of developing cancer/dysplasia. However, as for the population, the hypothesis that IIM had a higher risk of cancer/dysplasia than CIM was statistically confirmed only in the western population [pooled RR = 4.65 (95% CI 2.30–9.42)] but not in the East Asian population [pooled RR = 4.01 (95% CI 0.82–19.61)]. On the one hand, the number of studies included conducted in Asian countries was relatively small, and the result should be interpreted with caution. On the other hand, other than intestinal metaplasia subtypes, factors such as gene/race, preservative food consumption [34], and *Helicobacter pylori* infection [38] can also affect the occurrence of cancer/dysplasia. As an area of high incidence of gastric cancer, gene/race and diet may play a more important role in the malignant transformation of patients with intestinal metaplasia in East Asia which can interfere the results.

In addition to the comparison between IIM and CIM, this study also merged the risk of cancer/dysplasia of the three intestinal subtypes. The results showed that type III IM had a higher risk of cancer/dysplasia compared with type II + type I, or type II only. And type II also had a higher risk when compared with type I. Previous studies mainly underlined IIM and CIM. This study suggested that the risk of gastric cancer/dysplasia increased from type I, II, to III intestinal metaplasia. Thus, further attention should be paid on type III intestinal metaplasia in clinical practice. Correa’s gastric cancer model shows a gradual process from atrophy, intestinal metaplasia, to dysplasia and cancer. Is it possible that the type I, II, and III intestinal metaplasia representing different stages of a gradually developing process of gastric lesions? Further researches are needed to answer this question.

There were several limitations in this study. First, the results of some subgroup might be less accurate for the relatively small number of studies included. Second, we failed to explain the source of heterogeneity. Third, confounding factors were not controlled in all of the included studies. Besides, follow up time of cohort studies might affect the incidence of gastric cancer/dysplasia for patients with GIM.

## Conclusion

In conclusion, patients with IIM were at higher risk for gastric cancer/dysplasia than those with CIM. The risk of progressing to cancer/dysplasia in type I, II, and III intestinal metaplasia increased gradually. The predictive value of IIM for cancer/dysplasia in East Asian population needs further discussion.

## Supplementary Information

**Additional file 1: Figure S1.** Dysplasia/cancer risk among patients with IIM when compared with CIM: sensitivity analysis. **Figure S2.** Dysplasia/cancer risk among patients with IIM when compared with CIM: Egger’s publication bias plot (a), funnel plot (b), and filed funnel plot (c) after adding 5 more studies (inside the box) by trim and fill method. **Figure S3.** Forest plots for dysplasia/cancer risk among patients with type II IM when compared with type I IM (before sensitivity analysis). **Figure S4.** Dysplasia/cancer risk among patients with type II IM when compared with type I IM: sensitivity analysis. **Table S1.** Dysplasia/cancer risk among patients with IIM when compared with CIM: sensitivity analysis. **Table S2.** Dysplasia/cancer risk among patients with IIM when compared with CIM: publication bias (Egger’s test). **Table S3.** Dysplasia/cancer risk among patients with IIM when compared with CIM: sensitivity analysis: trim and fill method. **Table S4.** Dysplasia/cancer risk among patients with type II IM when compared with type I IM: sensitivity analysis.

## Data Availability

The datasets used or analyzed during the current study are available from the corresponding author on reasonable request.
