# Supplementary material for: A meta-analysis and systematic review on subtypes of gastric intestinal metaplasia and neoplasia risk
Source: Cancer Cell Int. 2021 Mar 17;21:173. doi: 10.1186/s12935-021-01869-0 (PMC7968216; doi:10.1186/s12935-021-01869-0)
Supplement: Supplementary file 1 — Additional file 1: Figure S1. Dysplasia/cancer risk among patients with IIM when compared with CIM: sensitivity analysis. Figure S2. Dysplasia/cancer risk among patients with IIM when compared with CIM: Egger’s publication bias plot (a), funnel plot (b), and filed funnel plot (c) after adding 5 more studies (inside the box) by trim and fill method. Figure S3. Forest plots for dysplasia/cancer risk among patients with type II IM when compared with type I IM (before sensitivity analysis). Figure S4. Dysplasia/cancer risk among patients with type II IM when compared with type I IM: sensitivity analysis. Table S1. Dysplasia/cancer risk among patients with IIM when compared with CIM: sensitivity analysis. Table S2. Dysplasia/cancer risk among patients with IIM when compared with CIM: publication bias (Egger’s test). Table S3. Dysplasia/cancer risk among patients with IIM when compared with CIM: sensitivity analysis: trim and fill method. Table S4. Dysplasia/cancer risk among patients with type II IM when compared with type I IM: sensitivity analysis. [file 12935_2021_1869_MOESM1_ESM.docx]

**
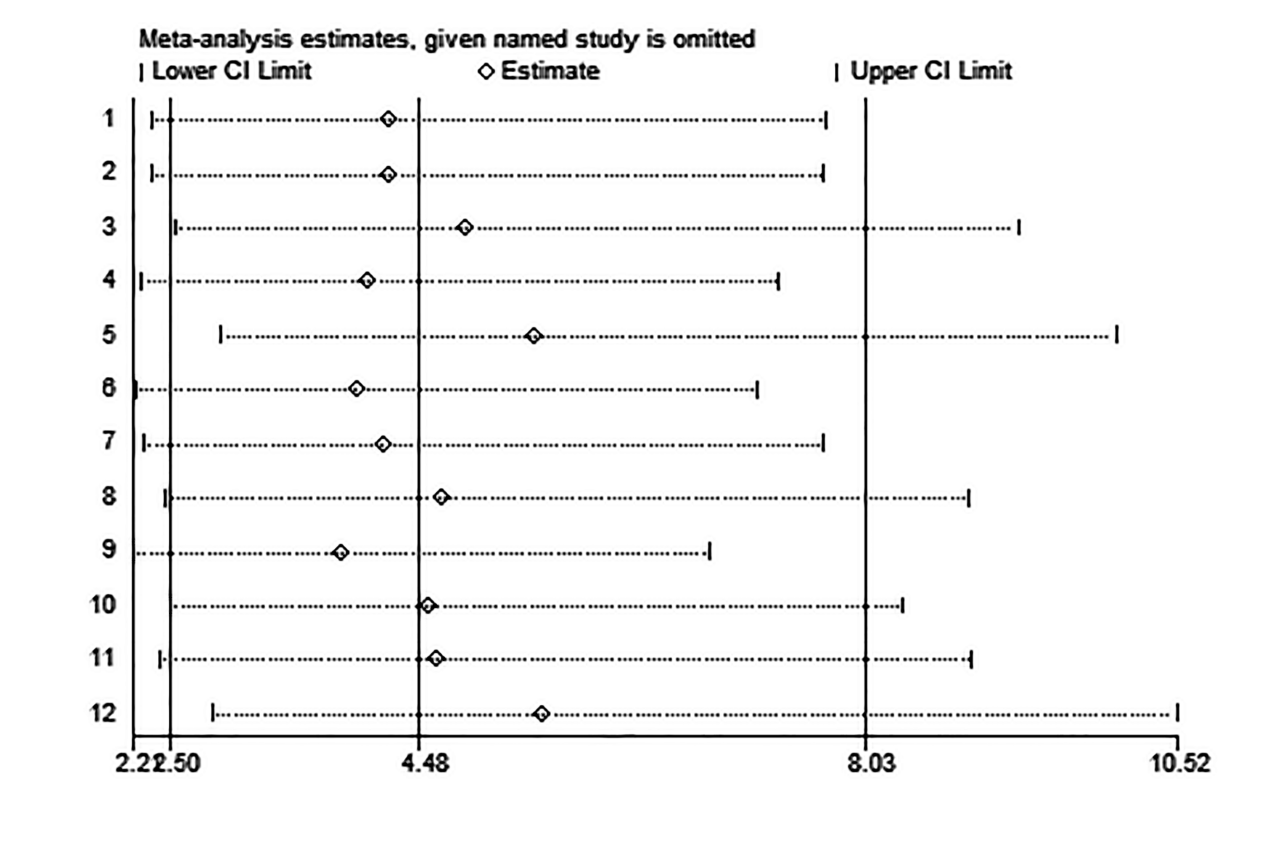
**

**Figure S1.** Dysplasia/cancer risk among patients with IIM when compared with CIM: sensitivity analysis.

**
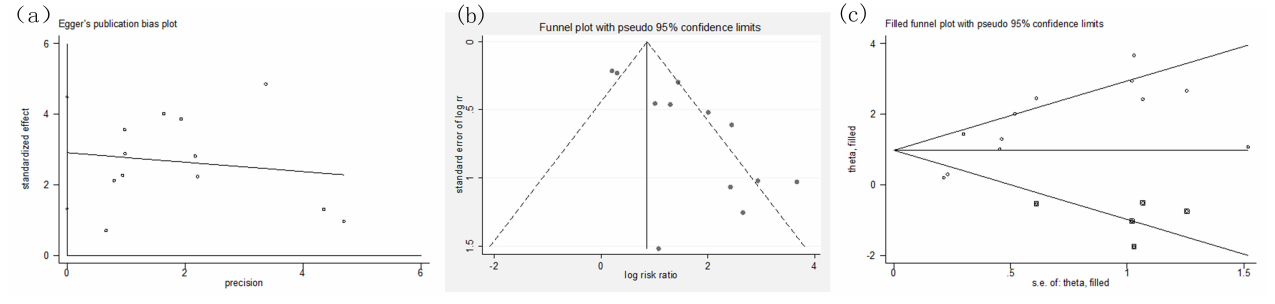
**

**Figure S2.** Dysplasia/cancer risk among patients with IIM when compared with CIM: Egger’s publication bias plot (a), funnel plot (b), and filed funnel plot(c) after adding 5 more studies (inside the box) by trim and fill method.

**Figure S3.** Forest plots for dysplasia/cancer risk among patients with type II IM when compared with type I IM (before sensitivity analysis).


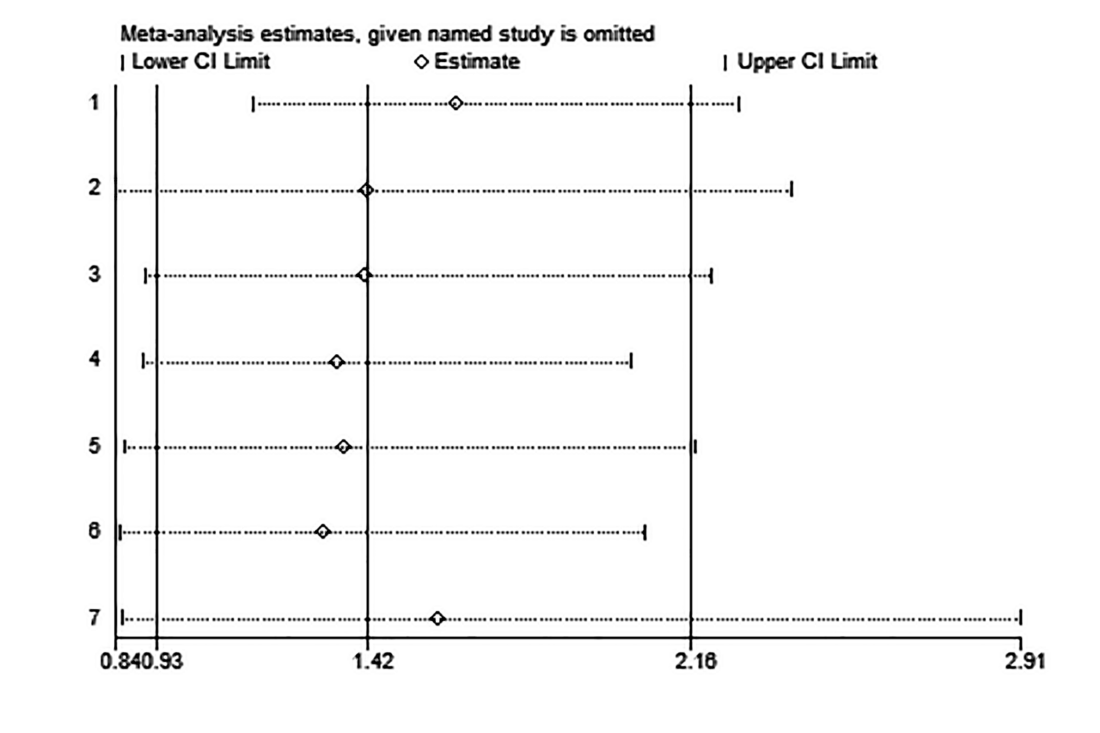


**Figure S4.** Dysplasia/cancer risk among patients with type II IM when compared with type I IM: sensitivity analysis.

**Table S1.** Dysplasia/cancer risk among patients with IIM when compared with CIM: sensitivity analysis.

| **Study omitted** | **Estimate** | **[95% Conf. Interval]** | |
| --- | --- | --- | --- |
| 1 | 4.2599592 | 2.3497031 | 7.7232113 |
| 2 | 4.2599254 | 2.3579731 | 7.6960011 |
| 3 | 4.8604665 | 2.5525551 | 9.2550917 |
| 4 | 4.0910387 | 2.2819293 | 7.3344073 |
| 5 | 5.4102325 | 2.9153223 | 10.040268 |
| 6 | 3.9994516 | 2.229542 | 7.1743946 |
| 7 | 4.2087851 | 2.2989712 | 7.7051301 |
| 8 | 4.6777091 | 2.4718034 | 8.8522263 |
| 9 | 3.8772688 | 2.2097368 | 6.8031688 |
| 10 | 4.5719614 | 2.5104167 | 8.3264389 |
| 11 | 4.6343923 | 2.417491 | 8.8842487 |
| 12 | 5.464726 | 2.8388612 | 10.51944 |
| Combined | 4.4824573 | 2.502451 | 8.0290978 |

**Table S2.** Dysplasia/cancer risk among patients with IIM when compared with CIM: publication bias (Egger's test).

| **Std_Eff** | **Coef.** | **Std. Err.** | **t** | **P>\|t\|** | **[95% Conf. Interval]** | |
| --- | --- | --- | --- | --- | --- | --- |
| slope | -.1334982 | .2890785 | -0.46 | 0.654 | -.7776052 | .5106088 |
| bias | 2.907853 | .7079506 | 4.11 | 0.002 | 1.330441 | 4.485266 |

**Table S3.** Dysplasia/cancer risk among patients with IIM when compared with CIM: sensitivity analysis: trim and fill method.

| **Before trim and fill method** | | | | | | | **After trim and fill method** | | | | | | |
| --- | --- | --- | --- | --- | --- | --- | --- | --- | --- | --- | --- | --- | --- |
| **Method** | **Pooled RR** | **95% CI** | | **Asymptotic** | | **No. Of studies** | **Method** | **Pooled RR** | **95% CI** | | **Asymptotic** | | **No. Of studies** |
|  | | **Lower** | **Upper** | **z_value** | **p_value** |  |  | | **Lower** | **Upper** | **z_value** | **p_value** |  |
| **Fixed** | 2.372 | 1.883 | 2.988 | 7.327 | <0.001 | 12 | **Fixed** | 2.080 | 1.666 | 2.596 | 6.469 | <0.001 | 17 |
| **Random** | 4.482 | 2.502 | 8.029 | 5.044 | <0.001 |  | **Random** | 2.664 | 1.546 | 4.591 | 3.530 | <0.001 |  |

**Table S4.** Dysplasia/cancer risk among patients with type II IM when compared with type I IM: sensitivity analysis.

| **Study omitted** | **Estimate** | **[95% Conf. Interval]** | |
| --- | --- | --- | --- |
| 1 | 1.6185008 | 1.1552687 | 2.2674768 |
| 2 | 1.4145175 | .83824575 | 2.386961 |
| 3 | 1.4122201 | .90579295 | 2.2017896 |
| 4 | 1.3480884 | .8999626 | 2.0193529 |
| 5 | 1.3644542 | .85971808 | 2.165518 |
| 6 | 1.31813 | .8468163 | 2.0517635 |
| 7 | 1.5765162 | .85391533 | 2.9105968 |
| Combined | 1.4171046 | .9313212 | 2.156276 |
